# Diabetes incidence in Austria: The role of famines on diabetes and related NCDs

**DOI:** 10.1016/j.heliyon.2023.e17570

**Published:** 2023-06-28

**Authors:** Michaela Kaleta, Michael Leutner, Stefan Thurner, Alexander Kautzky, Gottfried Endel, Noemi Kiss, Martin Robausch, Alexandra Kautzky-Willer, Peter Klimek

**Affiliations:** a Section for Science of Complex Systems, CeMSIIS, Medical University of Vienna, Vienna, Austria; b Complexity Science Hub Vienna, Vienna, Austria; c Gender Medicine Unit, Clinical Division of Endocrinology and Metabolism, Department of Medicine III, Medical University of Vienna, Vienna, Austria; d Santa Fe Institute, Santa Fe, NM, USA; e Clinical Division for Social Psychiatry, Department for Psychiatry and Psychotherapy, Medical University of Vienna, Vienna, Austria; f Austrian Social Insurance (Dachverband der Sozialversicherungen), Vienna, Austria; g Austrian Health Insurance Fund (Österreichische Gesundheitskasse), St. Pölten, Austria; h Gender Institute, Gars am Kamp, Austria

**Keywords:** Diabetes, Incidence, Register-based study, Famine, Perinatal exposure, Thrifty phenotype hypothesis, Intrauterine programming, Sex

## Abstract

Undernutrition in early life associates with increased risk for type 2 diabetes in later life. Whether similar associations hold for other diseases remains unclear. We aim to quantify how perinatal exposure to famines relates to the risk of becoming incident with type 2 diabetes in later life. Using population-wide medical claims data for Austrians aged >50y, yearly diabetes incidence was measured in an epidemiological progression model. We find incidence rates that increase from 2013 to 2017 and observe two famine-related birth cohorts of 5,887 patients with incidence rate increases for diabetes of up to 78% for males and 59% for females compared to cohorts born two years earlier. These cohorts show increased risks for multiple other diagnoses as well. Public health efforts to decrease diabetes must not only focus on lifestyle factors but also emphasize the importance of reproductive health and adequate nutrition during pregnancy and early postnatal life.

## 1 Introduction

The number of patients with diabetes mellitus is continuously rising. Several factors, such as sex, gender, overweight, obesity, lifestyle or genetic disposition have been identified as risk-modifying factors in the development of diabetes [1]. In addition undernutrition during early life results in higher risk of type 2 diabetes [2].

In several countries including Austria [3], China [4], or the Netherlands [5] a nontrivial age dependence was observed in the diabetes prevalence related to birth cohorts born in years of famine. In Austria, the risk for diabetes increased by a factor of more than two for people born during a famine when compared to cohorts born one year earlier or later [3]. Perinatal famine exposure was also associated with significantly increased risk for proliferative diabetic retinopathy in the Ukraine and China [6]. Such effects can be explained by intrauterine programming [5], where the metabolism of the (unborn) child pre-adapts to a nutritionally poor environment. If the expectation of poor nutrition does not materialize in later life (as was the case in Austria which experienced strong economic growth in the second half of the 20th century), these birth cohorts are of increased risk for metabolic and cardiovascular diseases [7,8].

First estimates for the yearly diabetes incidence in Austria have recently become available through an administrative dataset that covers 99.9% of the Austrian population, the LEICON dataset. This dataset identifies diabetes patients by means of prescriptions of antidiabetic medications and regular HbA1c measurements [[9], [10], [11]]. In particular, for 2015 an incidence rate of 626 per 100,000 of the population was found in Austria for individuals aged older than 14 years [11].

In this work we seek to understand whether there are famine-related birth cohort effects in diabetes incidence (and not only prevalence, as has already been established [3]) in Austria. We measure incidence rates based on a compartmental illness-death model for chronic diseases [[12], [13], [14]] that we adapted to the requirements of the LEICON data by keeping track of patients that discontinue and recontinue their antidiabetic treatments. Furthermore, we aim to quantify whether the famine-related diabetes cohort shows a different profile in terms of comorbid diagnoses and medications compated to a non-famine-related diabetes population.

## 2 Research design and methods

### 2.1 Data description

A pseudonymized research dataset provided by the Austrian Health Insurance (Dachverband der Österreichsichen Sozialversicherungen) is used. The data is provided by the LEICON group within the Austrian Health Insurance Fund (Österreichische Gesundheitskasse) [[9], [10], [11]]. Based on the population of insured patients in Austria, diabetes patients aged older than 50y and younger than 100y were identified via one of the inclusion criteria of receiving antidiabetic drug prescriptions, blood glucose or HbA1c measurements in the time period between January 1st, 2012 and December 31st, 2017.

For this population we obtained information on date of birth and death, sex and district of residence as well as date and dose of all antidiabetic medications (ATC codes starting A10) and HbA1c measurements. The LEICON dataset further contains information on main and side diagnoses (10th revision of the International Statistical Classification of Diseases and Related Health Problems (ICD-10)) associated with hospital stays during the observation period. General population information was taken from the Austrian statistical office (Statistik Austria).

### 2.2 Patient and public involvement

Research questions and outcome measures were identified in close collaboration with multiple stakeholders from social insurances in Austria. We particularly received input in the definition of inclusion and exclusion criteria for the anonymized research dataset as well as for the desired level of granularity in the results such that they provide actionable information for health planners.

### 2.3 Measuring incidence rates in an epidemiological model

Age- and sex-specific incidence rates were measured on the level of political districts (administrative regions with a mean population of 77,000) by means of an epidemiological compartmental model; the NIDEX model. For a full description of this model, see SI. In brief, the model categorizes the population into being either non-diabetic, incident, diabetic, or deceased in each year. Non-diabetic patients have not ever met any of the inclusion criteria for the diabetes group. Incident patients are those that meet one of the inclusion criteria for the first time in the given year. As long as they keep fulfilling at least one criterion in the following years, they remain in the diabetic compartment. If, however, they do not fulfill any criteria despite having been categorized as incident, they move to the erratic compartment. Upon continuation of diabetes therapies or blood glucose/HbA1c measurements, they move from the erratic to the diabetic compartment. From any of these states, patients transition to the deceased compartment upon death. The model ensures that patients that temporarily discontinue their diabetes treatments are not wrongly counted as incident upon reinitiation of their treatments. Incidence rates for a specific district, age group and sex are defined as the number of incident diabetes patients divided by the size of the general population which is not in a diabetic or erratic state.

### 2.4 Cohort definitions

**Fig. 1:**
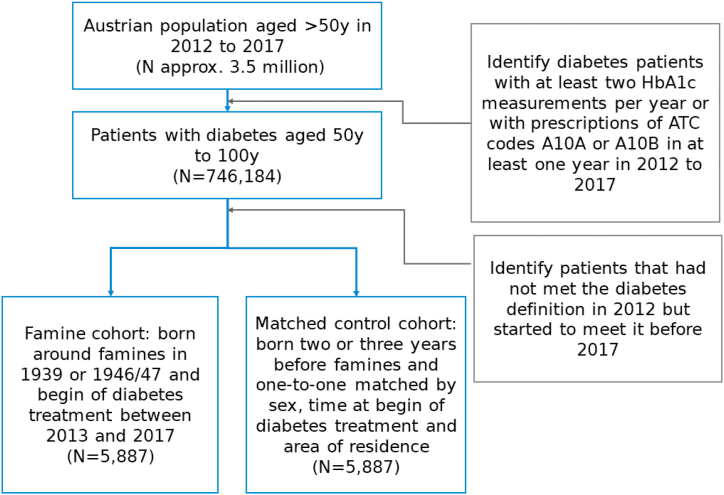
Summary of cohort construction. Patients with diabetes treatment are identified from the general population of about 3.5 million people aged 50y to 100y are identified. From these, patients born around famines who started diabetes treatment during the observation period are identified along with matched controls.

Using the NIDEX model we analyzed possible effects of nation-wide famines on diabetes incidence in Austria. Within our dataset, for people aged 50 to 100 years the two most notable famines took place in 1939 and 1946/1947 [3]. To assess the effects of famines on diabetes incidence rates we defined two cohorts for each famine: the famine cohort comprising people born during 1939 or 1946/1947, and the control cohort consisting of people born two to three years prior to the famines (1936 to 1937, or 1943 to 1944). Individuals born one year before the famine were not included as controls to have a clearer separation between famine and non-famine periods (the exact onset times of the famines are not known, hence some famine-related effects or precursors thereof could have an impact already one year earlier). Furthermore, to compare baseline characteristics of the combined famine cohorts (from both famine periods) and their corresponding controls, cases were matched 1:1 with controls based on age, sex and federal state. The construction of the famine cohort and its control group is also summarized in Fig. 1. Note that the control group is slightly older than the cases, meaning that one would expect increased prevalence of age-related chronic disorders amongst the controls. We adjusted for multiple testing using the Benjamini Hochberg correction controlling the false discovery rate at 0.01. Differences between famine and control group are reported as odds ratios (OR) with the 95% confidence intervals (CIs).

### 2.5 Regional famine effects

Regional differences in incidence rates between famine cohorts and control cohorts were assessed using district-level, sex-averaged harmonic means of incidence rates over all years (2013 to 2017). We calculated the famine effect on incidence as a relative difference, $\frac{\Delta_{\alpha }}{\alpha_{pre}}$, between the mean incidence rate of people born during famine years ($\alpha_{famine}$) and mean incidence rate of people born two to three years prior to famine ($\alpha_{pre}$). Birth years were assessed relative to the harmonic mean of the years 2013 to 2017. The district-level relative change in incidence rates is calculated as $\frac{\Delta_{\alpha }}{\alpha_{pre}}=\frac{\alpha_{famine}-\alpha_{pre}}{\alpha_{pre}}\text{.}$.

Ethics approval and consent to participate was not applicable as this is a modelling study based on the re-use of an anonymized research dataset. All features that would allow the identification of individuals have been sufficiently aggregated to make such an identification only possible with disproportionate and unrealistic efforts. The Federal Law on Documentation in the Health Care System in Austria provides the legal basis for this study: It allows the documentation of health-related data in the intra- and extramural outpatient and inpatient care sectors, as well as for the processing of patients' and service providers' data in pseudonymized form for certain purposes including (long-term) monitoring of epidemiological developments relevant to health policy as well as the implementation and further development of integrated health structure planning and health services research.

## 3 Results

### 3.1 Excess incidence rates related to famines

Out of approximately 3.5 million people in Austria aged 50y-100y, 746,184 people with diabetes treatments can be identified, see Fig. 1. Out of those, 5,887 patients were born around famines and started their treatments during the observation period.

**Fig. 2:**
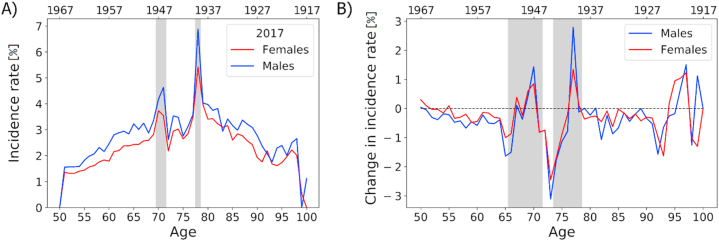
A) Age-specific incidence rates for patients aged between 50 and 100 for females and males in 2017. Birth years are shown on the second (top) x-axis. Results from the NIDEX model adjusting for patients with discontinued antidiabetic treatments. Grey vertical areas indicate patient cohorts that were born in years of famine. (B) Change in incidence rates over the years 2013 to 2017 for patients of ages 50 to 100. Red and blue lines highlight results from the NIDEX model for females and males, respectively. A general trend of decreased incidence rates in the NIDEX model can be seen, with two age ranges of increased incidence rates that can be linked with famine cohorts. (For interpretation of the references to colour in this figure legend, the reader is referred to the Web version of this article.)

Age-and sex-specific incidence rates of the NIDEX model for 2017 are shown in Fig. 2A). Incidence rates are higher in males than in females in almost all years and ages. In general, incidence increases from around 1% (female) and 1.5% (male) at age 50 to a maximum of around 5% (female) to 7% (male) at ages around 75. After this maximum, incidence declines with an increase in age. The age-specific incidence rates also show two distinct peaks on top of these age-dependent trends described above. A first peak can be seen for patients aged 78, born in 1939, and a second peak for patients aged around 70, born 1946/1947. These two birth cohorts align with years of famines in Austria, as shown by the grey vertical areas in Fig. 1A). Incidence in the famine cohort for 1939 increases for males from 3.9% to 6.9% (+78%) from the control to the famine cohort; for females the increase is from 3.4% to 5.4% (+59%). For those born in 1946/47, incidence rates increase by 26% (22%) from 3.5% (3.0%) to 4.4% (3.6%) for males (females).

Supplemental Fig. S1 shows the age-specific incidence rates for 2013–2016. With each passing year, the famine peaks shift from left to right as the famine cohorts age, clearly indicating that these peaks are cohort-specific effects, and not age-specific effects.

Fig. 2B) shows the change in incidence rates between the years 2013 to 2017 for both females and males. Overall, there is a trend toward decreased diabetes incidence. On average, incidence rates are between 0.5% and 1% lower in 2017 compared to 2013. Exceptions to this general trend arise in age groups that relate to famine cohorts. The famine periods reveal themselves as negative peaks (when the incidence in 2013 was increased due to a famine but not the 2017 incidence) followed by a positive peak (driven by the 2017 famine cohort).

### 3.2 Famine cohort characteristics

A general description of the combined famine and control cohorts is shown in Table 1. The famine cohorts column describes all patients born during any of the two famines, the control cohorts column represents all patients born two or three years prior to the famines. Cohorts' medication and diagnoses characteristics are evaluated based on patients’ data between 2012 and 2017. Significant differences between selected features of cohorts are tested using Chi^2^-tests and presented in form of p-values. Features including less than 5 individuals in any cohort were not tested for significance.

**Table 1:** Matched comparison between famine cohort (patients born during any of the two famines) and control cohort (patients born two or three years prior to any of the two famines) for selected disorders (ICD codes) and medication (ATC codes). Asterisks denote associations that remain significant after adjusting for multiple testing.

	Famine cohorts N (%)	Control cohorts N (%)	OR (95% CI)	p-value
N	5887	5887		–
Mean age in 2012 (SD)	68 (3.6)	65 (3.9)		–
Females	3059 (52)	3059 (52)		–
Major depressive disorder (F32–F33)	98 (1.7)	85 (1.4)	1.16 (0.86–1.55)	0.38
Dementia (F03)	24 (0.41)	10 (0.17)	2.41 (1.15–5.04)	<0.05
Dementia in Alzheimer's disease (F00)	12 (0.2)	5 (0.08)		–
Arterial hypertension (I10)	995 [17]	878 [15]	1.16 (1.05–1.28)	<0.01*
Hyperlipidemia (E78.5)	174 (3.0)	149 (2.5)	1.17 (0.94–1.46)	0.18
Ischemic heart diseases (I20–I25)	395 (6.7)	346 (5.9)	1.15 (0.99–1.34)	0.069
Myocardial infarction (I21.09 + I21.3 + I25.2)	22 (0.37)	18 (0.31)	1.22 (0.66–2.28)	0.64
Stroke (I63, I64)	78 (1.3)	82 (1.4)	0.95 (0.70–1.30)	0.82
Heart failure (I50)	131 (2.2)	81 (1.4)	1.63 (1.23–2.16)	<0.001*
pAVK (I73.8 + I73.9)	73 (1.2)	68 (1.2)	1.07 (0.77–1.50)	0.74
Acute kidney failure, chronic kidney disease (N17–N19)	223 (3.8)	127 (2.2)	1.79 (1.43–2.23)	<10^−6^*
Fatty liver (K76.0)	69 (1.2)	51 (0.87)	1.36 (0.94–1.95)	0.12
Fibrosis and cirrhosis of liver (K74)	17 (0.29)	14 (0.24)	1.21 (0.60–2.47)	0.72
Nicotine dependency (F17)	69 (1.2)	63 (1.1)	1.10 (0.78–1.55)	0.67
Alcohol abuse (F10.1)	13 (0.22)	9 (0.15)	1.45 (0.62–3.38)	0.53
Dependence on renal dialysis (Z99.2)	0	1 (0.02)		–
Heart transplant status (Z94.1)	1 (0.02)	3 (0.05)		–
Kidney transplant status (Z94.0)	1 (0.02)	0		–
Liver transplant status (Z94.4)	2 (0.03)	0		–
St.p. surgery (Z98)	33 (0.56)	26 (0.44)	1.27 (0.76–2.13)	0.44
Osteoporosis (M80–M82)	87 (1.5)	73 (1.2)	1.19 (0.87–1.63)	0.31
Diseases of arteries (I70–I79)	180 (3.1)	185 (3.1)	0.97 (0.79–1.20)	0.84
Overweight and obesity (E66)	157 (2.7)	149 (2.5)	1.06 (0.84–1.32)	0.69
COPD (J44)	205 (3.5)	160 (2.7)	1.29 (1.05–1.59)	<0.05
Arthritis (M06)	20 (0.34)	21 (0.36)	0.95 (0.52–1.76)	1
Insulin (A10A)	137 (2.3)	136 (2.3)	1.01 (0.79–1.28)	1
Oral antidiabetics (A10B)	1462 [25]	1570 [27]	0.91 (0.84–0.99)	<0.05
Biguanides (A10BA)	963 [16]	1098 [19]	0.85 (0.78–0.94)	<0.01*
Sulfonylureas (A10BB)	183 (3.1)	165 (2.8)	1.11 (0.90–1.38)	0.36
Comb. Of oral blood glucose lowering drugs (A10BD)	273 (4.6)	276 (4.7)	0.99 (0.83–1.17)	0.94
Alpha glucosidase inhibitors (A10BF)	1 (0.02)	8 (0.14)		–
Thiazolidinediones (A10BG)	20 (0.34)	30 (0.51)	0.67 (0.38–1.17)	0.21
DPP4-inhibitors (A10BH)	225 (3.8)	180 (3.1)	1.26 (1.03–1.54)	<0.05
GLP-1 analogues (A10BJ)	10 (0.17)	3 (0.05)		–
SGLT2-inhibitors (A10BK)	75 (1.3)	96 (1.6)	0.78 (0.57–1.06)	0.13
Other blood gluc. Lowering drugs, excl. insulins (A10BX)	6 (0.1)	4 (0.07)		–
Fibrates (C10AB)	110 (1.9)	149 (2.5)	0.73 (0.57–0.94)	<0.05
Lipid modifying agents (C10A)	3011 (51)	2880 (49)	1.09 (1.02–1.18)	<0.05
Statins (C10AA)	2918 (50)	2776 (47)	1.10 (1.02–1.18)	<0.01*
ARB (C09CA)	1010 (17)	913 (16)	1.13 (1.02–1.24)	<0.05
(C09AA)	972 (17)	946 (16)	1.03 (0.94–1.14)	0.54
Beta blocking agents (C07)	2001 (34)	1830 (31)	1.14 (1.06–1.23)	<0.001*
Aspirin (B01AC06)	542 (9.2)	490 (8.3)	1.12 (0.98–1.27)	0.097
Proton pump inhibitors (A02BC)	1711 (29)	1558 (26)	1.14 (1.05–1.23)	<0.01*
Aldosterone antagonists (C03DA)	360 (6.1)	303 (5.2)	1.20 (1.03–1.40)	<0.05
Clopidogrel (B01AC04)	378 (6.4)	344 (5.8)	1.11 (0.95–1.29)	0.25
Ticagrelor (B01AC24)	51 (0.87)	39 (0.66)	1.31 (0.86–1.99)	0.25
Prasugrel (B01AC22)	15 (0.25)	35 (0.59)	0.43 (0.23–0.78)	<0.01*
Antidepressants (N06A)	1270 (22)	1233 (21)	1.04 (0.95–1.13)	0.42
Glucocorticoids (H02AB09)	4 (0.07)	4 (0.07)	1.00 (0.25–4.00)	–
Corticosteroids (H02)	946 (16)	901 (15)	1.06 (0.96–1.17)	0.27

Baseline characteristics were evaluated based on information recorded between 2012 and 2017.

The famine cohort showed significantly increased frequency of arterial hypertension (17% vs. 15%, OR 1.16, 95% CI 1.05–1.28), heart failure (2.2% vs. 1.4%, OR 1.63, CI 1.23–2.16), and acute kidney failure and chronic kidney disease (3.8% vs. 2.2%, OR 1.79, CI 1.43–2.23). The famine cohort also showed different medication use with less frequent biguanides (16% vs. 19%, OR 0.85, CI 0.78–0.94), but more frequent dispensals of statins (50% vs. 47%, OR 1.10, 1.02–1.18), beta-blocking agents (34% vs. 31%, OR 1.14, CI 1.06–1.23), and proton pump inhibitors (29% vs. 26%, OR 1.14, CI 1.05–1.23). Results in the famine cohorts for increased dementia (0.41% vs 0.17%, OR 2.41, 1.15–5.04), COPD (3.5% vs. 2.7%, OR 1.29, CI 1.05–1.59), for less dispensals of oral antidiabetics (25% vs. 27%, OR 0.91, CI 0.84–0.99), fibrates (1.9% vs. 2.5%, OR 0.73, CI 0.57–0.94), and prasugrel (0.25% vs. 0.59%, OR 0.43, CI 0.23–0.78) but more frequent dispensals of DPP4-inhibitors (3.8% vs. 3.1%, OR 1.26, CI 1.03–1.54), ARB (17% vs. 16%, OR 1.13, CI 1.02–1.24), and aldosterone antagonists (6.1% vs. 5.2%, OR 1.20, CI 1.03–1.40) did not remain significant after adjusting for multiple testing.

We inquired the extent to which these results change when considering the two famine cohorts from 1939 to 1946/47 independently with their controls. This analysis shows that the results for acute kidney failure, chronic kidney disease and heart failure can be replicated in each famine cohort individually, though low case numbers render these results less conclusive.

### 3.3 Regional famine effects

**Fig. 3:**
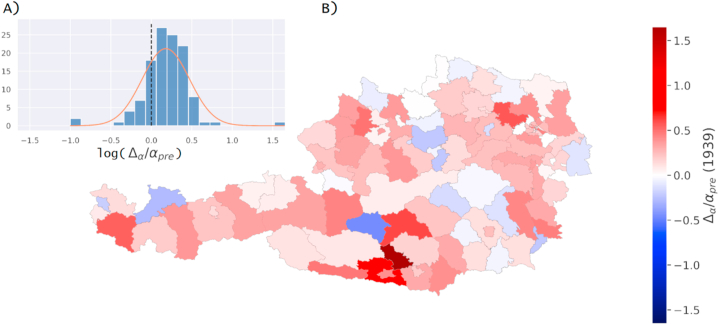
Regional effects of famine in the year 1939 measured as relative change in incidence rates. Results are shown as (A) a histogram inlay with distribution of relative change in incidence rate values and (B) on a map of political districts in Austria.

**Fig. 4:**
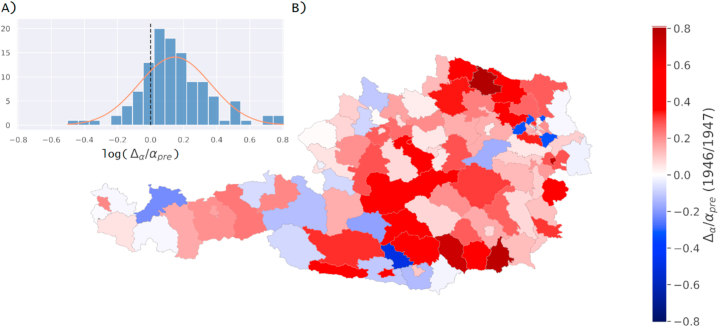
Regional effects of famine in years 1946/1947 measured as relative change in incidence rates. Results are shown as (A) a histogram inlay with distribution of relative change in incidence rate values and (B) on a map of political districts in Austria.

Regional differences in incidence rates between the famine cohort and control cohort were assessed for both famines individually on a district level averaged over sex. The resulting relative differences in incidence rates are shown in Fig. 3A,B (famine in 1939) and Fig. 4A,B (famine in 1946/1947) as (A) histograms and (B) on a map of political districts of Austria. Values for districts with zero incidence rate in famine years were coloured white (Wien-Umgebung in 1939, Rust (Stadt) in both 1939 and 1946/1947, Eisenstadt (Stadt) in 1939).

## 4 Discussion

Differences in prevalence related to birth year and respective famines during intrauterine and early life years in Austria have been previously described [3]. In addition, for the first time, temporal trends of three cohort-specific peaks in diabetes incidence rates can be identied, with larger peaks in men than in women, except for the oldest subgroup. These peaks correspond to birth years of famines and is in line with previously reported diabetes excess risks in the Austrian population [3]. Therefore, this new modeling analysis confirmed the finding that nutrition restriction in utero or early life is related to increased diabetes risk in adulthood [4]. Thus, gene-environment interactions due to famines lead to a subsequent intergenerational risk of diabetes resulting in epidemic states in the countries concerned. In the Dutch Hunger Winter and the Chinese Famine, long-term outcomes included higher rates of diabetes, obesity and hypertension in offspring of mothers which had been subjected to poor nutrition during pregnancy and complex maternal-placental interplay [4,5]. Also, children born during the Ukraine Famine showed a dose-response relationship between severity of famine during early development and probability of diabetes in adulthood [15], next to an 1.8 fold increase risk for diabetic retinopathy [6]. A 1.5 fold higher risk was seen in those exposed during early gestation. Sequelae of famines related to World War II were also documented in Ukraine [6] and some Asian countries during occupation, possibly contributing to their actual diabetes epidemic [16]. Among the many possible mechanisms are changes in DNA methylation of genes involved in growth, inflammation, glucose and lipid metabolism that occur as a result of in utero malnutrition, as reported in the Dutch Famine, with even time and sex-specific effects [17]. In China, also in non-diabetic adults famine in foetal and childhood-life was related to beta cell dysfunction [18]. Possibly, the combination of early-life undernutrition and postpartum overnutrition and development of obesity is highly predictive of diabetes in later life [19]. A recent metaanalysis of observational studies showed that foetal and childhood exposure to famine were associated with higher risks of cardiometabolic conditions in adulthood, with stronger effects for in-utero exposure and for women [20]. Although some studies show diabetes risk modifications after correction for lifestyle factors and cardiovascular health metrics [7], others find consistent results regardless of further adjustments [8]. In addition, increased risk of cardiovascular diseases were reported from China following early life famine exposure [21].

In the present study we could also find a higher occurrence of comorbidities, such as heart failure, arterial hypertension, COPD or kidney disease. Accordingly, a higher rate of hypertension was reported in adults born during the Biafran or the Dutch famines [4,5,22]. Fetal-infant exposure to the Biafran famine related to elevated systolic (+7 mmHg) and diastolic (+5 mmHg) blood pressure in later life [22]. A metaanalysis, studying the fetal origins hypothesis, confirmed a weak association between birthweight and subsequent blood pressure [23]. Strong evidence comes from animal studies showing that early nutrition affects the renin-angiotensin-system and arterial distensibility [24]. There is also some evidence of higher risk of dyslipidemia in later life as a consequence of intrauterine malnutrition-related metabolic programming [25,26]. The metabolic syndrome which was consistently described more frequently in subjects with intrauterine malnutrition may underlie their higher cardiometabolic risk including heart failure. Again, low birth weight was related with the risk of cardiovascular disease in adulthood [25].

The relationship of famine and increased COPD risk has been discussed earlier. The so called autophagy hypothesis claims, that several circumstances such as starvation induce pronounced autophagy processes which in further consequence lead to adaptation processes that are related to COPD [27]. Our findings about a higher risk of CKD in famine populations are in line with the Chinese famine study [28]. There is evidence that low birth weight is closely related to a lower kidney mass and that famine populations are characterized by a lower nephron number and kidney volume [29].

Increased risk for neurodegenerative as well as mental health disorders in cohorts exposed to famines were previously described. Nutritional deficits in the fetal period may accelerate aging of the brain and consequently cognitive decline, demonstrated by shorter telemore length at birth, unfavorable epigenetic changes relevant to neurogenesis and -plasticity, central nervous inflammation as observed in preclinical models as well as in cohorts born during the great Chinese famine 1959 to 61 or the Dutch famine 1944 to 45 [[30], [31], [32]]. Along these lines, similar aging-related mechanisms may also mediate higher occurrence of depression, among other mental health adversities. In contrast to results reported in Chinese subjects exposed to famines in utero [33,34], only marginally increased rates of major depression were observed here. This may be owed the comparatively old age of Austrian famine cohorts, considering that depressive symptoms are less frequently endorsed by elderly patients and rather attributed as inevitable challenges of old age by themselves as well as health professionals [35].

Altogether, molecular mechanisms linking fetal undernutrition and NCDs at older age may comprise endocrine effects including the hypothalamus-pituitary axis, glucocorticoids and growth hormone, epigenetic effects, oxidative stress, alterations in the renin-angiotensin system, sodium transporters and sympathetic activity [36].

The more pronounced multimorbidity status and hence the higher occurrence of kidney disease in the famine cohorts, could be the reason for the lower prescription rate of oral antidiabetics such as biguanides, although the latter finding might be confounded by our choice of the control cohort (see the limitations below). Interestingly the famine cohorts also showed a higher relative risk for dementia in the present study. The higher prescription rate of statins could indicate a higher occurrence of vascular disease in this specific cohort, which is the reason for vascular dementia [37].

Based on all these studies, overall public health efforts to decrease diabetes must not only focus on lifestyle factors but also emphasize the importance of reproductive health and adequate nutrition during pregnancy and early postnatal life.

A strength of our study is the large and comprehensive dataset on which it is based. The data covers 99.9% of the Austrian population over 2012–2017. This high coverage allows us to directly measure age-specific incidence rates for the entire population without introducing statistical uncertainties due to subsampling, without any additional modelling assumptions as it is usually the case in, e.g., regression-model-based approaches.

However, these data also have limitations. Information is not available for periods before 2012 and after 2017, so we could not ask whether famine-related effects were also observed in those years. Not all persons living in a particular district in 2012–2017 were also born in that district, which further dilutes the measurement of famine-related effects. Using data from the Austrian Statistical Office, we confirmed that 67% of the Austrian population lived in the same federal state where they were born in 2012; see also previous analyses of the contribution of internal migration to famine-related diabetes excess risks in Austria [3]. This means that the majority of the population can be expected to live close to the region where they might have been exposed to famine. However, due to the lack of data before 2012, the contribution of birth cohort-specific effects other than famine exposure to our results cannot be excluded. We identified diabetes patients based on dispensals of antidiabetic medications and number of HbA1c measurements only. Information on their blood glucose or HbA1c levels were not available, neither was diagnoses information from extramural care or outpatient visits available in Austria. Prescriptions for medications below a cost of EUR 4,70 were also not contained in the data, which, however, should not effect the antidiabetics considered in this work. There was also no information on socio-economic indicators or other clinical parameters, such as body mass index, smoking status, quality of glycemic control, and lifestyle factors like nutrition and exercise.

There was no information available on diagnoses or medications of patients without diabetes, hence it is not possible to compare famine-born diabetes patients with non-diabetic controls. Instead, we used diabetes patients born two years before the famines as controls. Consequently, the control cohort is slightly older than the famine cohort. In particular for age-related chronic diseases and their associated medications one might expect higher frequencies in the control than in the famine cohort. In contrast to this expectation, we find increased risks for several diagnoses in the famine cohort, suggesting that famine-related excess risks more than offset these age-related differences.

In conclusion, incidence of type 2 diabetes strongly depends on age, sex, place of residency and famine-related birth cohort effects.

## Declarations

### Author contribution statement

Michaela Kaleta, Alexandra Kautzky-Willer, Peter Klimek: Conceived and designed the experiments; Analyzed and interpreted the data; Wrote the paper.

Michael Leutner, Stefan Thurner, Alexander Kautzky: Analyzed and interpreted the data.

Gottfried Endel: Conceived and designed the experiments; Analyzed and interpreted the data; Contributed reagents, materials, analysis tools or data.

Noemi Kiss: Analyzed and interpreted the data; Contributed reagents, materials, analysis tools or data.

Martin Robausch: Contributed reagents, materials, analysis tools or data.

### Data availability statement

The authors do not have permission to share data.

## Declaration of competing interest

The authors declare the following financial interests/personal relationships which may be considered as potential competing interests: Peter Klimek reports financial support was provided by Vienna Science and Technology Fund under MA16-045. Alexandra Kautzky-Willer reports a relationship with Eli Lilly and Company that includes: speaking and lecture fees. Alexandra Kautzky-Willer reports a relationship with Boehringer Ingelheim GmbH that includes: speaking and lecture fees. Alexandra Kautzky-Willer reports a relationship with Novo Nordisk Inc that includes: speaking and lecture fees.
