# Supplementary material for: Diabetes incidence in Austria: The role of famines on diabetes and related NCDs
Source: Heliyon. 2023 Jun 28;9(7):e17570. doi: 10.1016/j.heliyon.2023.e17570 (PMC10395033; doi:10.1016/j.heliyon.2023.e17570)
Supplement: Multimedia component 1 [file mmc1.docx]

# Supplementary Material


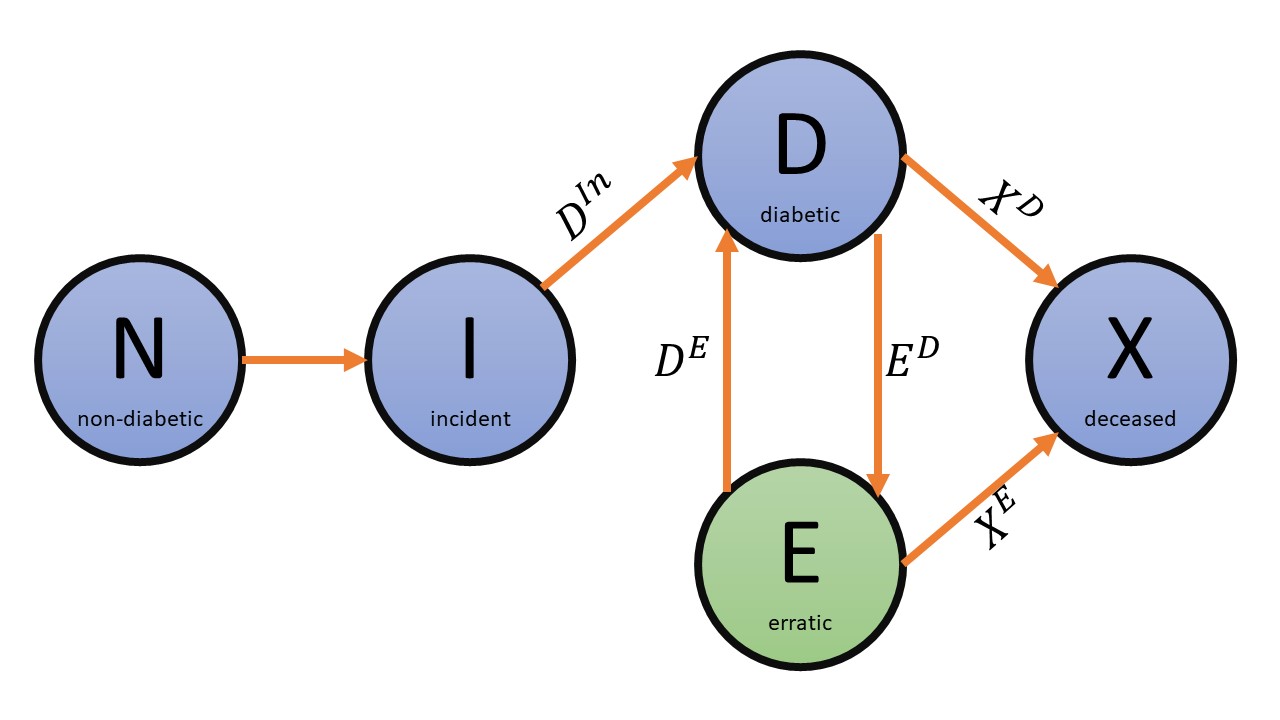


Figure S1. Overview of the NIDEX model to estimate diabetes incidence rates. Non-diabetic patients turn incident with a given rate and assume a diabetic state after receiving diabetes treatments. If these treatments are suspended for more than one year, they transition to the erratic state, from which they might return to the diabetic state if treatment is resumed. Patients die with different rates in the diabetic and erratic state.

## Modelling incidence rates

In the NIDEX model, see Figure S1, the total number of diabetes patients with age (or age groups) $a$, sex $g$ in year $t$, $D\left( a,t,g \right)$ is estimated based on the number of non-deceased diabetes patients in the last year, $D\left( a-1,t-1,g \right)$, in addition to all inflows to and outflows from the diabetic state. Inflows are the number of incident patients from the non-diabetic state, $D^{In}\left( a,t,g \right)$, and the erratic state, $D^{E}\left( a,t,g \right)$. Outflows are given by the number of excident patients, $E^{D}$, and the number of deceased diabetic patients, $X^{D}(a-1,t-1,g)$. This gives the following stock-flow equation for the age, sex and time dependent number of diabetic patients,

| $D\left( a,t,g \right)= D\left( a-1,t-1,g \right) + D^{In}\left( a,t,g \right) + D^{E}\left( a,t,g \right)-E^{D}\left( a,t,g \right)- X^{D}\left( a-1,t-1,g \right)$. | (1) |
| --- | --- |

The number of erratic patients can be expressed in a similar stock-flow equation. Using the number of deceased erratic patients, $X^{E}(a-1,t-1,g)$, we have

| $E\left( a,t,g \right)=E\left( a-1,t-1,g \right)- D^{E}\left( a,t,g \right)+ E^{D}\left( a,t,g \right)- X^{E}\left( a-1,t-1,g \right)$. | (2) |
| --- | --- |

From this model, dynamical age and sex dependent incidence rates can be derived. Let $N\left( a,t,g \right)$ be the total population of age *a* and sex *g* in year *t*. The diabetes incidence rate $\alpha\left( a,t,g \right)$ is the fraction of incident patients out of the general population which is not in a diabetic or erratic state,

| $\alpha\left( a,t,g \right)= D^{In}\left( a,t,g \right)/\left[ N\left( a-1,t-1,g \right)-D\left( a-1,t-1,g \right)-E(a-1,t-1,g) \right]$. | (3) |
| --- | --- |

Analogously, one could define an incidence rate $\alpha_{E}\left( a,t,g \right)$ from the erratic state, $\alpha_{E}\left( a,t,g \right)= D^{E}\left( a,t,g \right) / E\left( a-1,t-1,g \right)$, or an excidence rate $\beta\left( a,t,g \right)$ describing transitions from diabetic to erratic, $\beta\left( a,t,g \right)= E^{D}\left( a,t,g \right) / \left[ D\left( a-1,t-1,g \right)- X^{E}\left( a-1,t-1,g \right) \right]$. We do not explicitly model mortality of the general non-diabetic population.

To obtain age and sex standardized incidence rates [1], age groups of ten years for each sex were considered. Thereby the regional diabetes populations were standardized to the general Austrian population. Directly standardized rates $\alpha_{S}{(a,t,g)}_{B}$ are used to correct for effects of age, sex and others and are estimated for each district $B$ as $\alpha_{S}{(a,t,g)}_{B}=(\sum_{a,g} \frac{D^{In}\left( a,t,g \right)_{B}}{{P\left( a,t,g \right)}_{B}}*P\left( a,t,g \right))/(\sum_{a,g} P\left( a,t,g \right))$. The $\frac{D^{In}{(a,t,g)}_{B}}{{P(a,t,g)}_{B}}$ are the group specific rates of incident patients $D^{In}$ in the district population ${P(a,t,g)}_{B}$ and $P(a,t,g)$ is the total population of age $a$, sex $g$ in year $t$. We compare the study population in districts to the entire Austrian population as a standard population. Analogous standardisation is applied in the prevalence analysis by replacing the group specific incidence with prevalence.

## Age-specific incidence rates


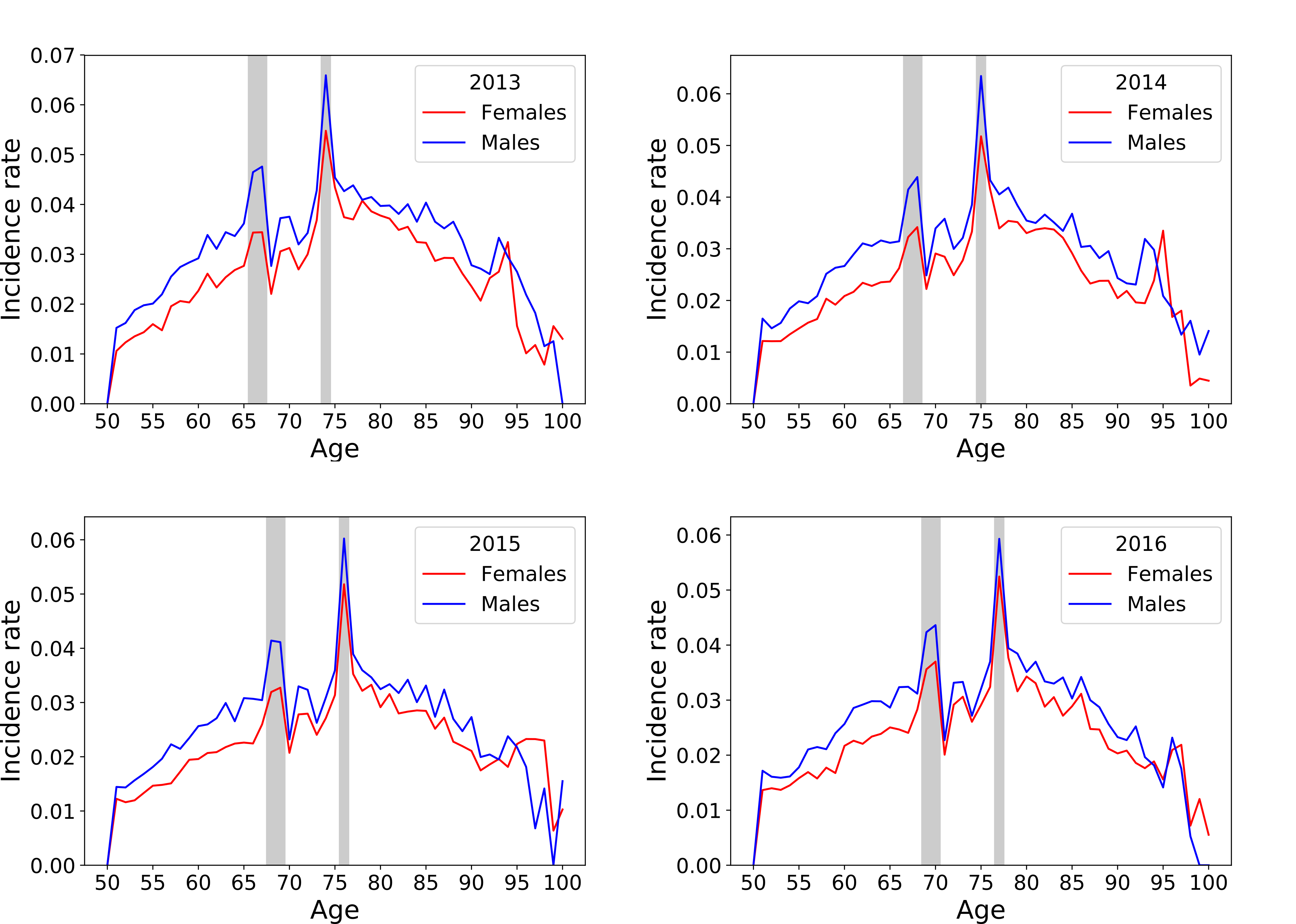


Figure S2. Age specific incidence rates for patients between 50 and 100 years old for females and males for years 2013 to 2016. Results from the NIDEX model adjusting for patients with discontinued antidiabetic treatments. Grey vertical areas indicate patient cohorts that were born in years of famine.

# References

[1] Schoenbach, Victor J., and Wayne D. Rosamond. Understanding the fundamentals of epidemiology: an evolving text. Chapel Hill: North Carolina (2000).
